# Brain size varies with temperature in vertebrates

**DOI:** 10.7717/peerj.301

**Published:** 2014-03-13

**Authors:** James F. Gillooly, Michael W. McCoy

**Affiliations:** 1 Department of Biology, University of Florida, Gainesville, FL, USA; 2 Department of Biology, East Carolina University, Greenville, NC, USA

**Keywords:** Encephalization, Allometry, Metabolic rate, Cranial capacity, Metabolic theory

## Abstract

The tremendous variation in brain size among vertebrates has long been thought to be related to differences in species’ metabolic rates. It is thought that species with higher metabolic rates can supply more energy to support the relatively high cost of brain tissue. And yet, while body temperature is known to be a major determinant of metabolic rate, the possible effects of temperature on brain size have scarcely been explored. Thus, here we explore the effects of temperature on brain size among diverse vertebrates (fishes, amphibians, reptiles, birds and mammals). We find that, after controlling for body size, brain size increases exponentially with temperature in much the same way as metabolic rate. These results suggest that temperature-dependent changes in aerobic capacity, which have long been known to affect physical performance, similarly affect brain size. The observed temperature-dependence of brain size may explain observed gradients in brain size among both ectotherms and endotherms across broad spatial and temporal scales.

## Introduction

Trends toward increasing relative brain size within or across groups have been identified in vertebrate evolution (Jerison, 1973), and many have argued that relatively large brains infer some form of evolutionary benefit (Dunbar & Shultz, 2007; Sol & Price, 2008; Kotrschal et al., 2013). However, the evolutionary benefit(s) is unclear since any direct link between brain size and intelligence remains inconclusive (Roth & Dicke, 2005). Moreover, there may be significant evolutionary costs that offset any such benefit, such as the relatively high energetic cost of maintaining brain tissue (Aiello & Wheeler, 1995). Still one thing is clear- the costs and benefits of larger brains have led to variation in vertebrate brain size that spans several orders of magnitude (Striedter, 2005).

In attempting to explain variation in relative brain size among vertebrates, many studies have suggested brain size is constrained by the energy made available through whole organism metabolism (Jerison, 1973; Martin, 1981). Support for this hypothesis is based in part on power law relationships of brain size with body size that are quite similar to those of metabolic rate (Jerison, 1973; Martin, 1981), thereby implying a nearly linear relationship between metabolic rate and brain size. But it remains unclear to what extent the similar body mass-scaling of brain size and metabolic rate may reflect energetic constraints on brain size (Isler & van Schaik, 2006). To begin, similarity in the scaling relationships does not take into account potential differences in the mass-specific energy use of brain tissue. Some have argued that neuron density, and thus mass-specific energy use of brains, varies with brain size (Macphail, 1982; Roth & Dicke, 2005). Secondly, such relationships do not consider possible tradeoffs between brain size and the size or energy use of other organs (Aiello & Wheeler, 1995). And finally, most studies linking brain size to metabolic rate have focused on endotherms (Isler & van Schaik, 2006), and do not address differences in brain size between ectotherms and endotherms (Jerison, 1973; Martin, 1981).

Much overlooked in this debate are the well-established effects of body temperature on whole organism metabolic rate (Krogh, 1916; Gillooly et al., 2001), and how such effects may influence brain size. If brain size is constrained by metabolic rate, then one might expect brain size to increase exponentially with temperature in the same way as metabolic rate. In principle, this is because higher temperature would increase energy supply by increasing biochemical reaction rates and associated dynamics (e.g., heart rate), and thus allow species to maintain more brain tissue. In the case of ectotherms, this would imply that brain size would vary systematically across species living in different thermal environments. While this would be surprising, changes in the mass of other organs with environmental temperature have been documented (Hammond, Szewczak & Król, 2001).

Thus, here we explore whether relative brain size (RB_m_; % body mass) varies with temperature after accounting for effects of body size. Specifically, we hypothesize that RB_m_ is proportional to mass-specific metabolic rate (*B*/*M*), and thus varies with body mass and temperature in the same way such that:

(1) \begin{eqnarray*} {\mathrm{RB}}_{\mathrm{m}}\propto B/M\propto {M}^{-1/4}{e}^{-E a/k T} \end{eqnarray*}

where *M*^−1/4^ describes the body-mass dependence of mass-specific metabolic rate, and *e*^−*Ea*/*kT*^ describes the temperature-dependence of metabolic rate. In the Boltzmann–Arrhenius term (i.e., *e*^−*E_a_*/*kT*^), *E_a_* is the average activation energy of the respiratory complex (−0.65 eV ), *k* is Boltzmann’s constant (8.62 × 10^−5^ eV K^−1^) (Gillooly et al., 2005), and *T* is absolute temperature in degrees Kelvin. We acknowledge that that the mechanistic basis of this expression remains unclear (Price et al., 2012), and that significant variation in the proposed size and temperature dependencies have been shown (White, Phillips & Seymour, 2006). Nonetheless, this expression provides a useful point of departure for examining the combined effects of body size and temperature on relative brain size.

Equation (1) predicts that the natural logarithm of temperature-corrected relative brain mass (i.e., ln(RB_m_ × *e*^*Ea*/*kT*^)) will scale linearly with the natural logarithm of body mass with a slope of about −1/4. As mentioned, this has already been shown for groups such as mammals (Jerison, 1973; Martin, 1981). More importantly, Eq. (1) also predicts that the natural logarithm of body mass-corrected relative brain mass (i.e., ln(RB_m_ × M^1/4^)) will be a linear function of inverse absolute temperature (i.e., 1/*kT*) with a slope of about −0.65. In other words, after accounting for body mass, Eq. (1) predicts relative brain mass will increase about 2.5 fold for every 10 °C increase in temperature (i.e., *Q*_10_ of 2.5; Gillooly et al., 2005). Implicit in these predictions is the assumption that mass-specific energy use in brains is approximately independent of brain size.

We examine the body size and temperature dependence of relative brain size using a dataset of 148 species from all major vertebrate groups (fishes, *n* = 31; amphibians, *n* = 11; reptiles, *n* = 18; birds, *n* = 39; and mammals, *n* = 58) over a body temperature range of about 40 °C. In the case of ectotherms, these body temperatures reflect the environmental temperatures in which the species naturally occur (see methods). The results point to an as yet unappreciated constraint on brain size in vertebrates—the effect of temperature.

## Methods

### Data

Data were collected from each taxonomic group in an effort to broadly represent the diversity in habitat, taxonomy, life history, body size and body temperature present in each group (Appendix S1).

Body temperatures were estimated using the resting body temperatures of endotherms (birds and mammals; Clarke & Rothery, 2008), and the average body or environmental temperature of ectotherms (amphibians, reptiles, and fishes). Thus, average environmental temperature was assumed to be equivalent to the average body temperature in ectotherms. Any differences in species average body temperature due to differences in activity level or other factors was therefore assumed to be small relative to the roughly 40 °C range in temperature. Body mass and brain mass data were taken mainly from the classic dataset of Crile & Quiring (1940). From this dataset, we included all species for which temperatures were available except one that appears to be in error (*Osmerus mordax*). We supplemented this dataset with additional sources if a particular species group (e.g., amphibians) or temperature range was underrepresented in the dataset. For amphibians, brain mass was estimated from brain volume assuming the density of water.

### Analyses

We evaluate the body mass and temperature dependence of relative brain mass across taxonomic groups. To partially account for possible effects of evolutionary relatedness among species, we first performed type II nested ANOVAS (Harvey & Pagel, 1991). With the nested ANOVAS, we examined the influence of taxonomic class, order within class, and family within order, to determine the appropriate level of analysis. We found that significant variation in all variables can be explained at the family level (*p* < 0.05), and thus performed all further analyses using mean values at this level.

Next, we used weighted multiple linear regression to estimate the body mass and temperature dependence of brain mass. Regressions were weighted depending on the proportion of taxa within each family. Regressions were fit based on a model of the form: lnRB_m_ = *a*ln*M* + *b*(1/*kT*) + *c*. Here *a* is a body-mass scaling exponent, *b* (*E_a_* in eV) characterizes the exponential temperature dependence, and *c* is a taxon-specific constant that includes random error. The variables *M* (g) and *T* (in Kelvin) in this formulation are mean values of body mass and temperature at the family level, and *k* is Boltzmann’s constant as defined above.

Finally, to address the differences in intercepts among taxonomic groups in estimating the overall relationship, we performed an ANOVA that allowed intercepts to vary across groups. We present the statistics for the observed body mass and temperature dependence of the overall relationship for both cases (i.e., fixed intercepts, intercepts allowed to vary).

To graphically represent the observed temperature-dependence of relative brain mass, we divided relative brain mass by the observed mass dependence (i.e., ln(RB_m_/*M^a^*)) based on multiple regression, and then plotted this “body-mass-corrected” value against inverse temperature (i.e., 1/*kT*). Similarly, to represent the observed body mass-dependence of relative brain mass, we divided relative brain mass by the observed temperature dependence (i.e., ln(RB_m_/*e*^−*Ea*/*kT*^)), and then plotted this “temperature-corrected” value against the natural logarithm of body mass. In this plot, then, the slope of the line represents *E_a_*, or the average activation energy from Eq. (1). While we recognize that brain mass does not have an activation energy per se, describing the temperature-dependence of brain mass in this way facilitates comparison to the temperature dependence of metabolic rate. Note too that body-mass corrected relative brain mass used here is roughly equivalent to what is often described as the “encephalization quotient” (Jerison, 1973).

## Results

**Figure 1:**
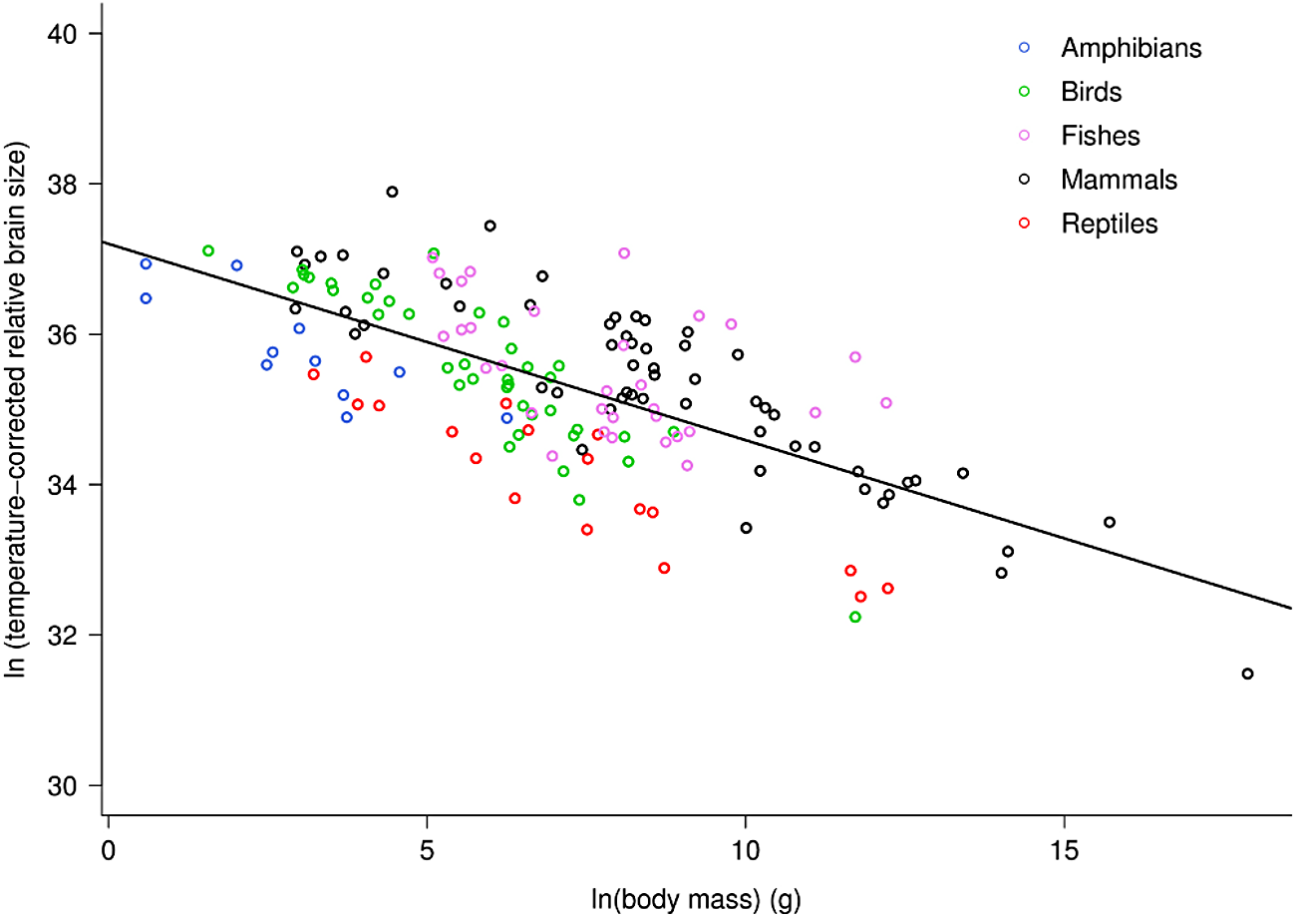
The natural logarithm of temperature-corrected relative brain size vs. the natural logarithm of body mass in vertebrates. Relative brain size is expressed as a percentage of body mass, body mass is expressed in grams, and temperature is in degrees Kelvin. The regression line shown is based on weighted values for data averaged at the level of family. Values are temperature-corrected using the Boltzmann–Arrhenius expression, as described in the methods. The equation for the fitted line is: ln (temp-corrected relative brain size) = −0.26*M* + ln(37.2).

Across vertebrates, multiple regression analysis indicated relative brain mass is related to body mass and temperature as: ln(RB_m_) = −0.26ln(*M*)−0.96(1/*kT*) + ln(37.2). Together, the two variables explained 75.5% of the variation in relative brain mass (RB_m_ range: 0.007–5.8%) for data analyzed at the level of family (Table S1; *F*_2,98_ = 150.9, *P* < 10^−15^). Both variables showed significant, independent effects on RB_m_ (both *P* < 10^−15^). This analysis showed that RB_m_ decreased with increasing body mass as RB_m_ ∝ *M*^−0.26^ (95% CI = 0.05) in agreement with model predictions (i.e., −0.25). Figure 1 shows a plot of the natural log of temperature-corrected relative brain size vs. the logarithm of body mass.

**Figure 2:**
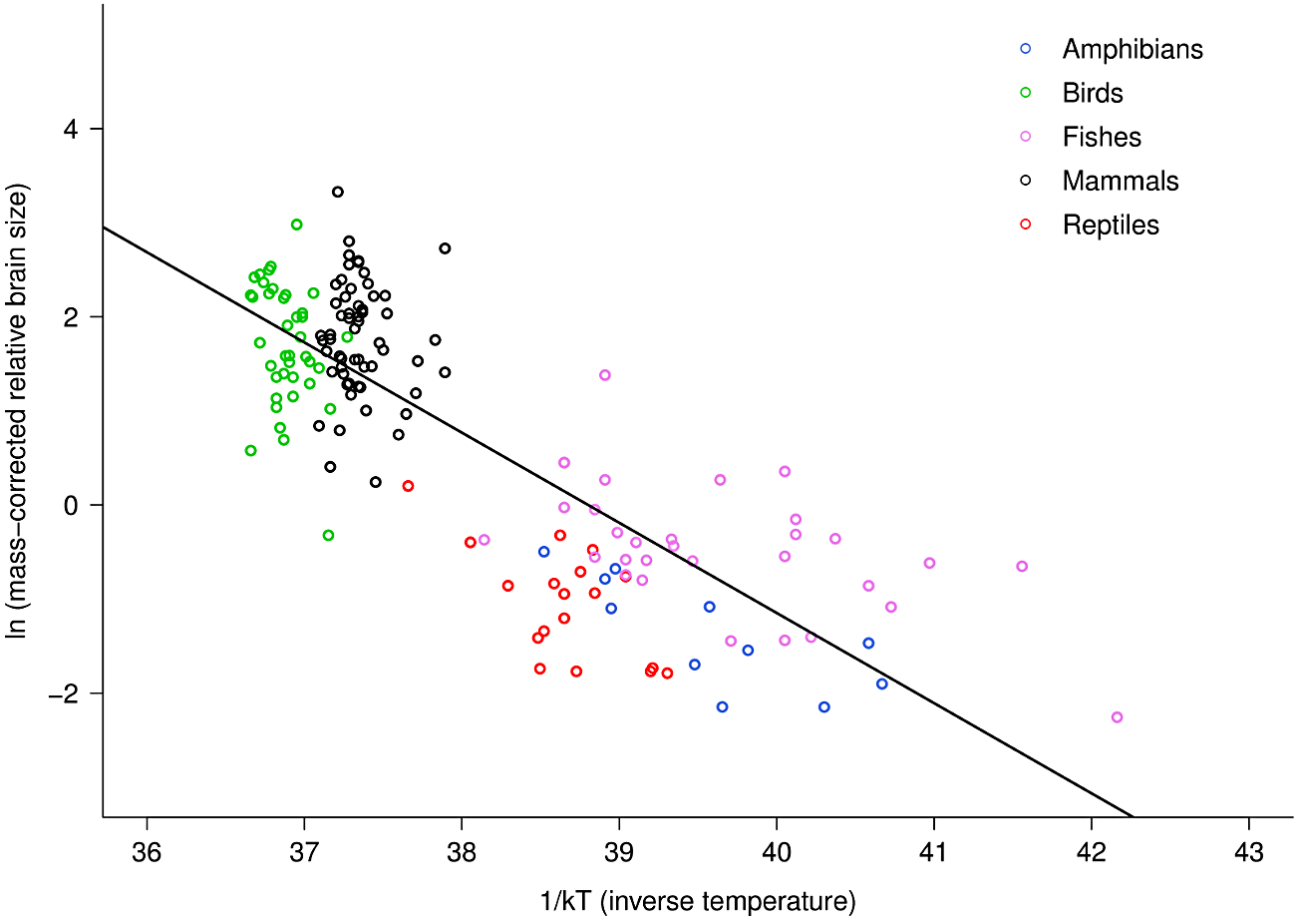
The natural logarithm of body mass-corrected relative brain size vs. inverse temperature in vertebrates. Relative brain size is expressed as a percentage of body mass, body mass is expressed in grams, and temperature is in degrees Kelvin (*T*). Inverse temperature is expressed using the Boltzmann–Arrhenius expression (1/*kT*), as described in the methods. The regression line shown is based on weighted values for data averaged at the level of family. Values are body mass-corrected based on results of multiple regression, as described in the methods. The equation for the fitted line is: ln (mass-corrected relative brain size) = −0.96(1/*kT*) + ln(37.2).

The multiple regression analysis also indicated a strong temperature dependence of RB_m_ after accounting for the effects of body mass. Figure 2 shows the natural logarithm of mass-corrected RB_m_ decreased with inverse temperature (1/*kT*) with a slope (−0.96; 95% CI = ± 0.13) that was significantly different from the predicted value of −0.65 (Table S1). Analyses at the species level, rather than the family level, yielded nearly identical results (Table S1).

Differences in intercept among taxonomic groups clearly affected the parameter estimates obtained using multiple regression because within and among group variation in body mass and temperature dependence were confounded. Across groups, intercepts varied by about 2.5 natural log units, or approximately 12-fold. Reptiles and fishes showed the lowest intercepts (17.52 and 17.75, respectively) fish were intermediate (18.85), and birds and mammals showed the highest intercepts (19.54 and 20.06, respectively). Allowing for differences in intercepts among groups thus provided different overall estimates for the size and temperature dependence of relative brain size. Specifically, this analysis yielded a body mass scaling exponent of −0.34, and an activation energy (temperature slope) of −0.47 (Table S1). In this case, the observed body mass dependence was significantly different than predicted (−0.34, 95% CI = −0.38 to −0.30) but the observed temperature dependence agreed with model predictions (−0.47, 95% CI = −0.69 to −0.26). Both had significant, independent effects on relative brain mass (see Table S1). The observed temperature dependence of −0.47 indicates that, on average, there is an eight-fold increase in relative brain mass from 0 to 40 °C across vertebrates after accounting for effects of body size. Within taxonomic groups, however, effects of temperature were only visible among the ectothermic groups, which had more variation in temperature (Fig. 2).

## Discussion

The results presented here provide support for the long-standing hypothesis that metabolic rate, and thus energy supply, constrains brain size. While the observed body mass-dependence within groups clearly varies, the overall dependence was similar to mass-specific metabolic rate, as previously described (Jerison, 1973; Martin, 1981). More surprisingly, relative brain size was shown to increase exponentially with temperature, albeit somewhat differently than model predictions. This suggests that temperature-dependent changes in aerobic capacity, which have long been known to affect physical performance (Bennett & Ruben, 1979), may similarly affect brain size.

Recognition of the temperature effects on brain size could provide insights into broad-scale spatial and temporal patterns in brain size for both ectotherms and endotherms. This is because environmental temperature affects not only the metabolic rate of ectotherms, but also that of endotherms, albeit to a lesser extent (Anderson & Jetz, 2005). For example, across space, one might expect gradients in brain size with elevation, latitude, or climate depending on the degree of temperature change and the taxonomic group in question. And across time, one might expect changes in brain size during the transition from water to land or the evolution of endothermy as these events involved changes in species’ temperatures and aerobic capacity (Bennett & Ruben, 1979). One could also speculate on the possibility of phenotypic plasticity in brain size with respect to temperature.

Still, even after accounting for differences in body size and temperature, there are large differences in brain size within and among groups. This highlights the fact that many factors combine to affect brain size and metabolism, not just body size and temperature. For example, many have recently pointed to ecological and social factors that may be important to brain size evolution (see Isler & van Schaik, 2006). Thus, these results may provide insights that help move us a step closer toward better understanding differences in vertebrate brain size.

## Supplemental Information

10.7717/peerj.301/supp-1 Table S1 Statistics describing body mass and temperature dependence of brain size Statistics evaluating body mass and temperature dependence of relative brain size across vertebrates using (A). simple multiple regression (MR), and (B). using ANOVA to allow for differences in intercepts among taxonomic groups. Analyses in panels (A) and (B) were performed at the level of family, to partially account for any effects of phylogenetic relatedness (see methods). Panel (C) shows results for MR for data at the species level. Data are provided in Appendix S1.

10.7717/peerj.301/supp-2 Appendix S1 Data and sources used to analyze effects of body mass (g) andtemperature (°C) on relative brain mass (% body mass) invertebrates
