# Supplementary material for: Brain size varies with temperature in vertebrates
Source: PeerJ. 2014 Mar 13;2:e301. doi: 10.7717/peerj.301 (PMC3961153; doi:10.7717/peerj.301)
Supplement: Table S1 — Statistics evaluating body mass and temperature dependence of relative brain size across vertebrates using (A). simple multiple regression (MR), and (B). using ANOVA to allow for differences in intercepts among taxonomic groups. Analyses in panels (A) and (B) were performed at the level of family, to partially account for any effects of phylogenetic relatedness (see methods). Panel (C) shows results for MR for data at the species level. Data are provided in Appendix S1. [file peerj-02-301-s001.docx]

Table 1: Statistics evaluating body mass and temperature dependence of relative brain size across vertebrates using **a.** simple multiple regression (MR), and **b.** using ANOVA to allow for differences in intercepts among taxonomic groups. Analyses in panels **a** and **b** were performed at the level of family, to partially account for any effects of phylogenetic relatedness (see methods). Panel **c** shows results for MR for data at the species level. Data are provided in Appendix 1.

| a. Statistics from MR fit to all data shown in Figures 1 and 2 | | | |
| --- | --- | --- | --- |
| Coefficients: | Estimate | 95% Conf. interval | |
| Intercept | 37.20 | 32.26 - 42.14 | |
| ln (M) | -0.26 | -0.31 - -0.21 | |
| 1/kT | -0.96 | -1.1 - -0.83 | |
| Adjusted R-squared: | 0.75 | : |  |
| F-statistic: | 150.9 | DF= 2, 98 | P < 2.20E-16 |

| b. Statistics from ANOVA, allowing group specific intercepts | | | | |
| --- | --- | --- | --- | --- |
| Coefficients: | Estimate | 95% Conf. interval | |  |
| Slopes |  |  | |  |
| ln (M) | -0.34 | -0.38 - -0.30 | |  |
| 1/kT | -0.47 | -0.69 - -0.26 | |  |
|  |  |  | |  |
| Intercepts |  |  | |  |
| Amphibians | 17.52 | 9.01 - 26.02 | |  |
| Birds | 19.54 | 10.31 - 28.78 | |  |
| Fishes | 18.85 | 9.89 - 27.83 | |  |
| Mammals | 20.06 | 10.88 - 29.33 | |  |
| Reptiles | 17.75 | 8.70 - 26.80 | |  |
|  |  |  |  |  |
| Adjusted R-squared: | 0.90 |  |  |  |
| F-statistic: | 144.2 | DF= 6, 98 | P < 2.20E-16 |  |

| c. Statistics from MR fit to data at species-level | | | |
| --- | --- | --- | --- |
| Coefficients: | Estimate | 95% Conf. interval | |
| Intercept | 37.16 | 33.05 - 41.26 | |
| ln (M) | -0.27 | -0.31 - -0.23 | |
| 1/kT | -0.96 | -1.06 - -0.85 | |
| Adjusted R-squared: | 0.74 |  |  |
| F-statistic: | 222.4 | DF= 2, 153 | P < 2.20E-16 |
