# Supplementary material for: Brain size varies with temperature in vertebrates
Source: PeerJ. 2014 Mar 13;2:e301. doi: 10.7717/peerj.301 (PMC3961153; doi:10.7717/peerj.301)
Supplement: Appendix S1 [file peerj-02-301-s002.docx]

Appendix 1: Data and sources used to analyze effects of body mass (g) and temperature (^o^C) on relative brain mass (% body mass) in vertebrates.

| **Class** | **Order** | **Family** | **Species** | **Body Mass** | |  | **Temperature** | | | | | **Rel. Brain Mass** | |  | **M** |  |
| --- | --- | --- | --- | --- | --- | --- | --- | --- | --- | --- | --- | --- | --- | --- | --- | --- |
| Mammalia | [Artiodactyla](http://en.wikipedia.org/wiki/Artiodactyla) | Bovidae | *Capra hircus* | 27660.0 | | [1] | | 39.5 | [2] | | 0.419 | | [1] | | | |
| Mammalia | [Artiodactyla](http://en.wikipedia.org/wiki/Artiodactyla) | Bovidae | *Raphicerus campestris* | 8620.0 | | [1] | | 39.0 | [2] | | 0.574 | | [1] | | | |
| Mammalia | [Artiodactyla](http://en.wikipedia.org/wiki/Artiodactyla) | Cervidae | *Odocoileus virginianus* | 65090.0 | | [1] | | 39.0 | [2] | | 0.323 | | [1] | | | |
| Mammalia | [Artiodactyla](http://en.wikipedia.org/wiki/Artiodactyla) | Cervidae | *Rangifer tarandus* | 128470.0 | | [1] | | 39.2 | [2] | | 0.238 | | [1] | | | |
| Mammalia | [Artiodactyla](http://en.wikipedia.org/wiki/Artiodactyla) | [Giraffidae](http://en.wikipedia.org/wiki/Giraffidae) | *Giraffa camelopardalis* | 1220000.0 | | [1] | | 38.5 | [2] | | 0.057 | | [1] | | | |
| Mammalia | [Artiodactyla](http://en.wikipedia.org/wiki/Artiodactyla) | [Hippopotamidae](http://en.wikipedia.org/wiki/Hippopotamidae) | *Hippopotamus amphibius* | 1351000.0 | | [1] | | 35.4 | [2] | | 0.053 | | [1] | | | |
| Mammalia | Carnivora | Canidae | *Canis latrans* | 8510.0 | | [1] | | 37.0 | [2] | | 0.990 | | [1] | | | |
| Mammalia | Carnivora | Canidae | *Canis lupus* | 29940.0 | | [1] | | 38.4 | [2] | | 0.508 | | [1] | | | |
| Mammalia | Carnivora | Canidae | *Canis mesomelas* | 2850.0 | | [1] | | 38.0 | [2] | | 1.614 | | [1] | | | |
| Mammalia | Carnivora | Canidae | *Vulpes lagopus* | 3385.0 | | [1] | | 38.4 | [2] | | 1.315 | | [1] | | | |
| Mammalia | Carnivora | Canidae | *Vulpes vulpes* | 4625.0 | | [1] | | 38.7 | [2] | | 1.152 | | [1] | | | |
| Mammalia | Carnivora | Felidae | *Acinonyx jubatus* | 22200 | | [1] | | 39 | [2] | | 0.110 | | [1] | | | |
| Mammalia | Carnivora | Felidae | *Felis bangsi* | 25960.0 | | [1] | | 37.5 | [2] | | 0.497 | | [1] | | | |
| Mammalia | Carnivora | Felidae | *Felis capensis* | 9955.0 | | [1] | | 37.5 | [2] | | 0.670 | | [1] | | | |
| Mammalia | Carnivora | Felidae | *Felis ocreata* | 2700.0 | | [1] | | 37.5 | [2] | | 1.056 | | [1] | | | |
| Mammalia | Carnivora | Felidae | *Panthera leo* | 190800.0 | | [1] | | 37.9 | [2] | | 0.135 | | [1] | | | |
| Mammalia | Carnivora | Felidae | *Panthera onca* | 34470.0 | | [1] | | 37.7 | [2] | | 0.426 | | [1] | | | |
| Mammalia | Carnivora | Felidae | *Panthera pardus* | 48000.0 | | [1] | | 37.7 | [2] | | 0.281 | | [1] | | | |
| Mammalia | Carnivora | Felidae | *Panthera tigris* | 209000.0 | | [1] | | 37.5 | [2] | | 0.144 | | [1] | | | |
| Mammalia | Carnivora | [Herpestidae](http://en.wikipedia.org/wiki/Herpestidae) | *Ichneumia albicauda* | 4400.0 | | [1] | | 39.4 | [2] | | 0.643 | | [1] | | | |
| Mammalia | Carnivora | Mustelidae | *Mustela erminea* | 1693.0 | | [1] | | 39.6 | [2] | | 0.333 | | [1] | | | |
| Mammalia | Carnivora | Odobenidae | *Odobenus rosmarus* | 667000 | | [1] | | 36.4 | [2] | | 0.169 | | [1] | | | |
| Mammalia | Carnivora | Phocidae | *Erignathus barbatus* | 281000 | | [1] | | 37.2 | [2] | | 0.164 | | [1] | | | |
| Mammalia | Carnivora | [Procyonidae](http://en.wikipedia.org/wiki/Procyonidae) | *Nasua narica* | 399.0 | | [1] | | 38.6 | [2] | | 5.840 | | [1] | | | |
| Mammalia | Carnivora | Procyonidae | *Potos flavus* | 2620.0 | | [1] | | 36.1 | [2] | | 1.185 | | [1] | | | |
| Mammalia | Carnivora | [Procyonidae](http://en.wikipedia.org/wiki/Procyonidae) | *Procyon lotor* | 5216.0 | | [1] | | 38.0 | [2] | | 0.819 | | [1] | | | |
| Mammalia | Carnivora | Ursidae | *Ursus arctos* | 142880.0 | | [1] | | 38.0 | [2] | | 0.164 | | [1] | | | |
| Mammalia | Carnivora | Ursidae | *Ursus maritimus* | 317000.0 | | [1] | | 36.8 | [2] | | 0.160 | | [1] | | | |
| Mammalia | Cetacea | Balaenopteridae | *Balaenoptera musculus* | 58059000 | | [1] | | 36.6 | [2] | | 0.012 | | [1] | | | |
| Mammalia | Chiroptera | Phyllostomidae | *Desmodus rotundus* | 28.0 | | [1] | | 37.3 | [2] | | 3.343 | | [1] | | | |
| Mammalia | [Cingulata](http://en.wikipedia.org/wiki/Cingulata) | Dasypodidae | *Dasypus novemcinctus* | 3701.0 | | [1] | | 34.5 | [2] | | 0.384 | | [1] | | | |
| Mammalia | [Didelphimorphia](http://en.wikipedia.org/wiki/Didelphimorphia) | [Didelphidae](http://en.wikipedia.org/wiki/Didelphidae) | *Didelphis marsupialis* | 1147.0 | | [1] | | 35.0 | [2] | | 0.418 | | [1] | | | |
| Mammalia | [Hyracoidea](http://en.wikipedia.org/wiki/Hyracoidea) | Procaviidae | *Heterohyrax brucei* | 750.0 | | [1] | | 36.7 | [2] | | 1.636 | | [1] | | | |
| Mammalia | [Lagomorpha](http://en.wikipedia.org/wiki/Lagomorpha) | [Leporidae](http://en.wikipedia.org/wiki/Leporidae) | *Lepus arcticus* | 2640.0 | | [1] | | 38.9 | [2] | | 0.527 | | [1] | | | |
| Mammalia | Pilosa | [Bradypodidae](http://en.wikipedia.org/wiki/Bradypodidae) | *Bradypus variegatus* | 3774.0 | | [1] | | 33.0 | [2] | | 0.477 | | [1] | | | |
| Mammalia | Pilosa | Cyclopedidae | *Cyclopes didactylus* | 86.0 | | [1] | | 33.0 | [2] | | 4.779 | | [1] | | | |
| Mammalia | Pilosa | [Megalonychidae](http://en.wikipedia.org/wiki/Megalonychidae) | *Choloepus hoffmanni* | 5271.0 | | [1] | | 34.4 | [2] | | 0.493 | | [1] | | | |
| Mammalia | Pilosa | [Myrmecophagidae](http://en.wikipedia.org/wiki/Myrmecophagidae) | *Tamandua tetradactyla* | 3692.0 | | [1] | | 33.5 | [2] | | 0.677 | | [1] | | | |
| Mammalia | Primates | Aotidae | *Aotus zonalis* | 8890.0 | | [1] | | 38.0 | [2] | | 1.327 | | [1] | | | |
| Mammalia | Primates | Cebidae | *Saimiri oerstedii* | 907.0 | | [1] | | 38.0 | [2] | | 2.789 | | [1] | | | |
| Mammalia | Primates | [Cercopithecidae](http://en.wikipedia.org/wiki/Cercopithecidae) | *Cercopithecus aethiops* | 3955.0 | | [1] | | 37.5 | [2] | | 1.540 | | [1] | | | |
| Mammalia | Primates | [Cercopithecidae](http://en.wikipedia.org/wiki/Cercopithecidae) | *Cercopithecus mitis* | 4550.0 | | [1] | | 37.5 | [2] | | 1.464 | | [1] | | | |
| Mammalia | Primates | [Cercopithecidae](http://en.wikipedia.org/wiki/Cercopithecidae) | *Papio cynocephalus* | 19510.0 | | [1] | | 37.2 | [2] | | 0.897 | | [1] | | | |
| Mammalia | Primates | [Galagidae](http://en.wikipedia.org/wiki/Galagidae) | *Galago senegalensis* | 200.0 | | [1] | | 37.9 | [2] | | 2.500 | | [1] | | | |
| Mammalia | [Proboscidea](http://en.wikipedia.org/wiki/Proboscidea) | [Elephantidae](http://en.wikipedia.org/wiki/Elephantidae) | *Loxodonta africana* | 6654000.0 | | [1] | | 36.2 | [2] | | 0.086 | | [1] | | | |
| Mammalia | [Rodentia](http://en.wikipedia.org/wiki/Rodent) | [Caviidae](http://en.wikipedia.org/wiki/Caviidae) | *Hydrochoerus isthmius* | 27670.0 | | [1] | | 37.1 | [2] | | 0.189 | | [1] | | | |
| Mammalia | [Rodentia](http://en.wikipedia.org/wiki/Rodent) | [Cricetidae](http://en.wikipedia.org/wiki/Cricetidae) | *Dicrostonyx groenlandicus* | 55.2 | | [1] | | 38.4 | [2] | | 1.522 | | [1] | | | |
| Mammalia | [Rodentia](http://en.wikipedia.org/wiki/Rodent) | [Cricetidae](http://en.wikipedia.org/wiki/Cricetidae) | *Microtus pennsylvanicus* | 41.3 | | [1] | | 38.5 | [2] | | 1.840 | | [1] | | | |
| Mammalia | [Rodentia](http://en.wikipedia.org/wiki/Rodent) | [Cricetidae](http://en.wikipedia.org/wiki/Cricetidae) | *Ondatra zibethicus* | 900.0 | | [1] | | 37.4 | [2] | | 0.592 | | [1] | | | |
| Mammalia | [Rodentia](http://en.wikipedia.org/wiki/Rodent) | [Dasyproctidae](http://en.wikipedia.org/wiki/Dasyproctidae) | *Dasyprocta punctata* | 3172.0 | | [1] | | 38.4 | [2] | | 0.578 | | [1] | | | |
| Mammalia | [Rodentia](http://en.wikipedia.org/wiki/Rodent) | [Dipodidae](http://en.wikipedia.org/wiki/Dipodidae) | *Zapus hudsonius* | 19.3 | | [1] | | 37.3 | [2] | | 3.575 | | [1] | | | |
| Mammalia | [Rodentia](http://en.wikipedia.org/wiki/Rodent) | [Erethizontidae](http://en.wikipedia.org/wiki/New_World_porcupine) | *Erethizon dorsatum* | 3410.0 | | [1] | | 37.5 | [2] | | 0.562 | | [1] | | | |
| Mammalia | [Rodentia](http://en.wikipedia.org/wiki/Rodent) | Muridae | *Lemmus trimucronatus* | 48.0 | | [1] | | 38.1 | [2] | | 1.310 | | [1] | | | |
| Mammalia | [Rodentia](http://en.wikipedia.org/wiki/Rodent) | Muridae | *Mastomys coucha* | 21.8 | | [1] | | 38.0 | [2] | | 3.257 | | [1] | | | |
| Mammalia | [Rodentia](http://en.wikipedia.org/wiki/Rodent) | [Sciuridae](http://en.wikipedia.org/wiki/Sciuridae) | *Tamias striatus* | 75.0 | | [1] | | 38.2 | [2] | | 2.960 | | [1] | | | |
| Mammalia | [Rodentia](http://en.wikipedia.org/wiki/Rodent) | [Sciuridae](http://en.wikipedia.org/wiki/Sciuridae) | *Tamiasciurus hudsonicus* | 248.0 | | [1] | | 38.7 | [2] | | 2.024 | | [1] | | | |
| Mammalia | Soricomorpha | Soricidae | *Blarina brevicauda* | 18.8 | | [1] | | 38.3 | [2] | | 1.872 | | [1] | | | |
| Mammalia | Soricomorpha | Talpidae | *Scalopus aquaticus* | 39.6 | | [1] | | 36.0 | [2] | | 2.929 | | [1] | | | |
|  |  |  |  |  | |  | |  |  | |  | |  | | |  |
| Aves | [Accipitriformes](http://en.wikipedia.org/wiki/Accipitriformes) | [Accipitridae](http://en.wikipedia.org/wiki/Accipitridae) | *Accipiter striatus* | 520.0 | | [1] | | 42.8 | [2] | | 1.096 | | [1] | | | |
| Aves | [Accipitriformes](http://en.wikipedia.org/wiki/Accipitriformes) | [Accipitridae](http://en.wikipedia.org/wiki/Accipitridae) | *Buteo buteo* | 558.0 | | [1] | | 40.5 | [2] | | 1.416 | | [1] | | | |
| Aves | [Accipitriformes](http://www.itis.gov/servlet/SingleRpt/SingleRpt?search_topic=TSN&search_value=823961) | [Cathartidae](http://www.itis.gov/servlet/SingleRpt/SingleRpt?search_topic=TSN&search_value=175263) | *Cathartes aura* | 494.0 | | [1] | | 39.9 | [2] | | 1.883 | | [1] | | | |
| Aves | [Anseriformes](http://en.wikipedia.org/wiki/Anseriformes) | [Anatidae](http://en.wikipedia.org/wiki/Anatidae) | *Anas acuta* | 670.0 | | [1] | | 41.5 | [2] | | 0.739 | | [1] | | | |
| Aves | [Anseriformes](http://en.wikipedia.org/wiki/Anseriformes) | [Anatidae](http://en.wikipedia.org/wiki/Anatidae) | *Anas carolinensis* | 305.0 | | [1] | | 41.2 | [2] | | 1.023 | | [1] | | | |
| Aves | [Anseriformes](http://en.wikipedia.org/wiki/Anseriformes) | Anatidae | *Mergus serrator* | 770.0 | | [1] | | 41.9 | [2] | | 0.687 | | [1] | | | |
| Aves | Charadriiformes | Laridae | *Larus argentatus* | 535.0 | | [1] | | 41.2 | [2] | | 0.950 | | [1] | | | |
| Aves | Charadriiformes | Laridae | *Larus delawarensis* | 720.0 | | [1] | | 41.3 | [2] | | 1.211 | | [1] | | | |
| Aves | [Charadriiformes](http://en.wikipedia.org/wiki/Charadriiformes) | Laridae | *Larus philadelphia* | 205.0 | | [1] | | 41.4 | [2] | | 1.215 | | [1] | | | |
| Aves | [Ciconiiformes](http://en.wikipedia.org/wiki/Ciconiiformes) | [Ciconiidae](http://en.wikipedia.org/wiki/Ciconiidae) | *Leptoptilos crumeniferus* | 7130.0 | | [1] | | 39.6 | [2] | | 0.423 | | [1] | | | |
| Aves | Columbiformes | Columbidae | *Columba livia* | 247.0 | | [1] | | 41.0 | [2] | | 0.923 | | [1] | | | |
| Aves | [Falconiformes](http://en.wikipedia.org/wiki/Falconiformes) | [Accipitridae](http://en.wikipedia.org/wiki/Accipitridae) | *Buteo jamaicensis* | 1029.0 | | [1] | | 40.6 | [2] | | 0.975 | | [1] | | | |
| Aves | [Falconiformes](http://en.wikipedia.org/wiki/Falconiformes) | [Accipitridae](http://en.wikipedia.org/wiki/Accipitridae) | *Haliaetus vocifer* | 3500.0 | | [1] | | 41.9 | [2] | | 0.369 | | [1] | | | |
| Aves | [Falconiformes](http://en.wikipedia.org/wiki/Falconiformes) | Falconidae | *Falco sparverius* | 112.0 | | [1] | | 40.5 | [2] | | 2.241 | | [1] | | | |
| Aves | [Galliformes](http://en.wikipedia.org/wiki/Galliformes) | [Phasianidae](http://en.wikipedia.org/wiki/Phasianidae) | *Gallus gallus* | 1263.0 | | [1] | | 41.5 | [2] | | 0.310 | | [2] | | | |
| Aves | [Galliformes](http://en.wikipedia.org/wiki/Galliformes) | [Phasianidae](http://en.wikipedia.org/wiki/Phasianidae) | *Lagopus lagopus* | 542.0 | | [1] | | 41.7 | [2] | | 0.439 | | [1] | | | |
| Aves | [Galliformes](http://en.wikipedia.org/wiki/Galliformes) | [Phasianidae](http://en.wikipedia.org/wiki/Phasianidae) | *Phasianus colchicus* | 625.0 | | [1] | | 41.9 | [2] | | 0.526 | | [1] | | | |
| Aves | [Gaviiformes](http://en.wikipedia.org/wiki/Charadriiformes) | Gaviidae | *Gavia stellata* | 1568.0 | | [1] | | 39.0 | [2] | | 0.407 | | [1] | | | |
| Aves | [Gruiformes](http://en.wikipedia.org/wiki/Gruiformes) | [Gruidae](http://en.wikipedia.org/wiki/Gruidae) | *Grus canadensis* | 165.1 | | [1] | | 40.8 | [2] | | 5.197 | | [1] | | | |
| Aves | [Passeriformes](http://en.wikipedia.org/wiki/Passerine) | [Corvidae](http://en.wikipedia.org/wiki/Corvidae) | *Corvus brachyrhynchos* | 337.0 | | [1] | | 42.2 | [2] | | 2.760 | | [1] | | | |
| Aves | [Passeriformes](http://en.wikipedia.org/wiki/Passeriformes) | [Emberizidae](http://en.wikipedia.org/wiki/Emberizidae) | *Melozpiza melodia* | 21.0 | | [1] | | 42.8 | [2] | | 5.238 | | [1] | | | |
| Aves | [Passeriformes](http://en.wikipedia.org/wiki/Passerine) | [Hirundinidae](http://en.wikipedia.org/wiki/Swallow) | *Hirundo rustica* | 21.5 | | [1] | | 41.4 | [2] | | 4.186 | | [1] | | | |
| Aves | [Passeriformes](http://en.wikipedia.org/wiki/Passerine) | [Icteridae](http://en.wikipedia.org/wiki/Icterid) | *Molothrus ater* | 66.0 | | [1] | | 42.3 | [2] | | 4.076 | | [1] | | | |
| Aves | [Passeriformes](http://en.wikipedia.org/wiki/Passerine) | [Icteridae](http://en.wikipedia.org/wiki/Icterid) | *Quiscalus quiscula* | 82.0 | | [1] | | 43.1 | [2] | | 3.561 | | [1] | | | |
| Aves | [Passeriformes](http://en.wikipedia.org/wiki/Passerine) | [Mimidae](http://en.wikipedia.org/wiki/Mimidae) | *Dumetella carolinensis* | 33.0 | | [1] | | 42.6 | [2] | | 4.273 | | [1] | | | |
| Aves | [Passeriformes](http://en.wikipedia.org/wiki/Passerine) | [Passeridae](http://en.wikipedia.org/wiki/Sparrow) | *Passer domesticus* | 23.3 | | [1] | | 42.1 | [2] | | 4.378 | | [1] | | | |
| Aves | [Passeriformes](http://en.wikipedia.org/wiki/Passeriformes) | Sturnidae | *Sturnus vulgaris* | 58.4 | | [1] | | 41.5 | [2] | | 3.116 | | [1] | | | |
| Aves | [Passeriformes](http://en.wikipedia.org/wiki/Passerine) | [Turdidae](http://en.wikipedia.org/wiki/Thrush_(bird)) | *Sialia sialis* | 34.0 | | [1] | | 42.3 | [2] | | 3.765 | | [1] | | | |
| Aves | [Passeriformes](http://en.wikipedia.org/wiki/Passerine) | [Turdidae](http://en.wikipedia.org/wiki/Thrush_(bird)) | *Turdus migratoris* | 69.3 | | [1] | | 43.2 | [2] | | 3.016 | | [1] | | | |
| Aves | [Passeriformes](http://en.wikipedia.org/wiki/Passeriformes) | [Tyrannidae](http://en.wikipedia.org/wiki/Tyrannidae) | *Sayornis phoebe* | 18.1 | | [1] | | 43.3 | [2] | | 4.365 | | [1] | | | |
| Aves | [Pelecaniformes](http://en.wikipedia.org/wiki/Pelecaniformes) | [Ardeidae](http://en.wikipedia.org/wiki/Ardeidae) | *Ardea alba* | 1030.0 | | [1] | | 40.1 | [2] | | 0.594 | | [1] | | | |
| Aves | [Pelecaniformes](http://en.wikipedia.org/wiki/Pelecaniformes) | [Ardeidae](http://en.wikipedia.org/wiki/Ardeidae) | *Ardea intermedia* | 525.0 | | [1] | | 40.1 | [2] | | 0.895 | | [1] | | | |
| Aves | [Pelecaniformes](http://en.wikipedia.org/wiki/Pelican) | [Pelecanidae](http://en.wikipedia.org/wiki/Pelican) | *Pelecanus occidentalis* | 3290.0 | | [1] | | 42.2 | [2] | | 0.530 | | [1] | | | |
| Aves | [Phoenicopteriformes](http://en.wikipedia.org/wiki/Phoenicopteriformes) | [Phoenicopteridae](http://en.wikipedia.org/wiki/Phoenicopteridae) | *Phoenicopterus minor* | 1483.0 | | [1] | | 41.0 | [2] | | 0.471 | | [1] | | | |
| Aves | [Procellariiformes](http://en.wikipedia.org/wiki/Procellariiformes) | [Procellariidae](http://en.wikipedia.org/wiki/Procellariidae) | *Puffinus griseus* | 268.0 | | [1] | | 40.3 | [2] | | 1.123 | | [1] | | | |
| Aves | [Strigiformes](http://en.wikipedia.org/wiki/Owl) | Strigidae | *Bubo virginianus* | 1177.0 | | [1] | | 40.8 | [2] | | 1.164 | | [1] | | | |
| Aves | Struthioniformes | Struthionidae | *Struthio camelus* | 123000.0 | | [1] | | 39.1 | [2] | | 0.034 | | [1] | | | |
| Aves | [Trochiliformes](http://en.wikipedia.org/wiki/Trochiliformes) | [Trochilidae](http://en.wikipedia.org/wiki/Trochilidae) | *Amazilia tzacatl* | 4.8 | | [1] | | 38.1 | [2] | | 3.958 | | [1] | | | |
|  |  |  |  |  | |  | |  |  | |  | |  | | | |
| Amphibia | Anura | Hylidae | *Hyla septentrionalis* | 12.0 | | [3] | | 24.7 | [4] | | 0.174 | | [5] | | |  |
| Amphibia | Anura | Hylidae | *Hyla versicolor* | | 13.2 | [6] | | 28.0 | [7] | | 0.310 | | [6] | | | |
| Amphibia | Anura | [Pipidae](http://en.wikipedia.org/wiki/Pipidae) | *Xenopus laevis* | | 20.0 | [8] | | 20.0 | [9] | | 0.155 | | [5] | | | |
| Amphibia | Anura | Ranidae | *Rana catesbeiana* | | 520.0 | [8] | | 25.0 | [10] | | 0.089 | | [5] | | | |
| Amphibia | Anura | Ranidae | *Rana clamitans* | | 96.3 | [6] | | 24.5 | [7] | | 0.154 | | [6] | | | |
| Amphibia | Anura | Ranidae | *Rana temporaria* | | 42.1 | [8] | | 19.4 | [11] | | 0.044 | | [5] | | | |
| Amphibia | Caudata | Ambystomatidae | *Ambystoma mexicanum* | | 40.0 | [3] | | 20.7 | [7] | | 0.070 | | [5] | | | |
| Amphibia | Caudata | [Plethodontidae](http://en.wikipedia.org/wiki/Lungless_salamander) | *Batrachoseps attenuatus* | | 1.8 | [8] | | 12.1 | [7] | | 0.128 | | [5] | | | |
| Amphibia | Caudata | [Plethodontidae](http://en.wikipedia.org/wiki/Lungless_salamander) | *Plethodon cinereus* | | 1.8 | [8] | | 18.2 | [7] | | 0.183 | | [5] | | | |
| Amphibia | Caudata | [Plethodontidae](http://www.amphibiaweb.org/lists/Plethodontidae.shtml) | *Desmognathus monticola* | | 7.5 | [8] | | 12.7 | [7] | | 0.136 | | [5] | | | |
| Amphibia | Caudata | [Plethodontidae](http://www.amphibiaweb.org/lists/Plethodontidae.shtml) | *Desmognathus quadramaculatus* | | 25.7 | [8] | | 14.7 | [7] | | 0.050 | | [5] | | | |
|  |  |  |  | |  |  | |  |  | |  | |  | | | |
| Reptilia | [Crocodylia](http://en.wikipedia.org/wiki/Crocodylia) | [Alligatoridae](http://en.wikipedia.org/wiki/Alligatoridae) | *Alligator mississippiensis* | | 205000.0 | [1] | | 22.8 | [12] | | 0.007 | | [1] | | |  |
| Reptilia | [Crocodylia](http://en.wikipedia.org/wiki/Crocodylia) | [Crocodylidae](http://en.wikipedia.org/wiki/Crocodylidae) | *Crocodylus americanus* | | 134000.0 | [1] | | 28.0 | [13] | | 0.012 | | [1] | | | |
| Reptilia | Squamata | [Boidae](http://en.wikipedia.org/wiki/Boidae) | *Boa imperator* | | 1829.0 | [1] | | 26.4 | [14] | | 0.024 | | [1] | | | |
| Reptilia | Squamata | [Colubridae](http://en.wikipedia.org/wiki/Colubrid) | *Coluber constrictor* | | 590.0 | [1] | | 28.3 | [15] | | 0.046 | | [1] | | | |
| Reptilia | Squamata | [Colubridae](http://en.wikipedia.org/wiki/Colubrid) | *Natrix natrix* | | 70.0 | [1] | | 27.5 | [16] | | 0.143 | | [1] | | | |
| Reptilia | Squamata | [Colubridae](http://en.wikipedia.org/wiki/Colubrid) | *Nerodia cyclopion* | | 220.0 | [1] | | 27.0 | [17] | | 0.095 | | [1] | | | |
| Reptilia | Squamata | [Colubridae](http://en.wikipedia.org/wiki/Colubridae) | *Thamnophis sirtalis* | | 57.0 | [1] | | 25.6 | [15] | | 0.216 | | [1] | | | |
| Reptilia | Squamata | [Helodermatidae](http://en.wikipedia.org/wiki/Helodermatidae) | *Heloderma suspectum* | | 514.0 | [1] | | 27.2 | [15] | | 0.142 | | [1] | | | |
| Reptilia | Squamata | [Iguanidae](http://en.wikipedia.org/wiki/Iguanidae) | *Amblyrhynchus cristatus* | | 4190.0 | [1] | | 27.0 | [18] | | 0.034 | | [1] | | | |
| Reptilia | Squamata | [Lacertidae](http://en.wikipedia.org/wiki/Lacertidae) | *Lacerta viridis* | | 50.0 | [1] | | 31.7 | [19] | | 0.242 | | [1] | | | |
| Reptilia | Squamata | [Phrynosomatidae](http://en.wikipedia.org/wiki/Phrynosomatidae) | *Phrynosoma coronatum* | | 25.0 | [1] | | 34.9 | [15] | | 0.528 | | [1] | | | |
| Reptilia | Squamata | [Pythonidae](http://en.wikipedia.org/wiki/Pythonidae) | *Python molurus* | | 6140.0 | [1] | | 28.2 | [20] | | 0.018 | | [1] | | | |
| Reptilia | Squamata | [Viperidae](http://en.wikipedia.org/wiki/Viperidae) | *Agkistrodon piscovoris* | | 728.0 | [1] | | 26.2 | [15] | | 0.088 | | [1] | | | |
| Reptilia | Testudines | [Cheloniidae](http://en.wikipedia.org/wiki/Cheloniidae) | *Chelonia mydas* | | 114300.0 | [1] | | 22.0 | [21] | | 0.008 | | [1] | | | |
| Reptilia | [Testudines](http://en.wikipedia.org/wiki/Testudines) | [Chelydridae](http://en.wikipedia.org/wiki/Chelydridae) | *Chelydra serpentina* | | 5125.0 | [1] | | 22.7 | [22] | | 0.019 | | [1] | | | |
| Reptilia | [Testudines](http://en.wikipedia.org/wiki/Testudines) | [Chelydridae](http://en.wikipedia.org/wiki/Chelydridae) | *Macrochelys temminckii* | | 1848.0 | [1] | | 25.5 | [23] | | 0.055 | | [1] | | | |
| Reptilia | [Testudines](http://en.wikipedia.org/wiki/Testudines) | [Emydidae](http://en.wikipedia.org/wiki/Emydidae) | *Clemmys guttatus* | | 2163.0 | [1] | | 24.0 | [24] | | 0.063 | | [1] | | | |
| Reptilia | [Testudines](http://en.wikipedia.org/wiki/Testudines) | [Testudinidae](http://en.wikipedia.org/wiki/Testudinidae) | *Testudo graeca* | | 320.0 | [1] | | 29.8 | [25] | | 0.094 | | [1] | | | |
|  |  |  |  | |  |  | |  |  | |  | |  | | | |
| Chondrichthyes | [Carcharhiniformes](http://en.wikipedia.org/wiki/Carcharhiniformes) | [Carcharhinidae](http://en.wikipedia.org/wiki/Carcharhinidae) | *Galeocerdo cuvier* | | 200000.0 | [1] | | 19.5 | [26] | | 0.054 | | [1] | | |  |
| [Chondrichthyes](http://en.wikipedia.org/wiki/Chondrichthyes) | [Carcharhiniformes](http://en.wikipedia.org/wiki/Carcharhiniformes) | [Carcharhinidae](http://en.wikipedia.org/wiki/Carcharhinidae) | *Prionace glauca* | | 65750.0 | [27] | | 16.5 | [28] | | 0.032 | | [27] | | | |
| [Chondrichthyes](http://en.wikipedia.org/wiki/Chondrichthyes) | Lamniformes | [Odontaspididae](http://en.wikipedia.org/wiki/Odontaspididae) | *Carcharias taurus* | | 123000.0 | [1] | | 16.5 | [29] | | 0.067 | | [1] | | | |
| [Chondrichthyes](http://en.wikipedia.org/wiki/Chondrichthyes) | [Myliobatiformes](http://en.wikipedia.org/wiki/Myliobatiformes) | [Dasyatidae](http://en.wikipedia.org/wiki/Dasyatidae) | *Dasyatis sabina* | | 17580.0 | [1] | | 25.0 | [30] | | 0.310 | | [1] | | | |
|  |  |  |  | |  |  | |  |  | |  | |  | | | |
| Actinopterygii | [Cypriniformes](http://en.wikipedia.org/wiki/Cypriniformes) | [Cyprinidae](http://en.wikipedia.org/wiki/Cyprinidae) | *Cyprinus carpio* | | 1061.0 | [1] | | 31.0 | [31] | | 0.112 | | [1] | | |  |
| Actinopterygii | [Esociformes](http://en.wikipedia.org/wiki/Esociformes) | [Esocidae](http://en.wikipedia.org/wiki/Esocidae) | *Esox lucius* | | 373.8 | [1] | | 23.5 | [31] | | 0.143 | | [1] | | | |
| Actinopterygii | [Gadiformes](http://en.wikipedia.org/wiki/Gadiformes) | Gadidae | *Gadus morhua* | | 10600.0 | [1] | | 10.0 | [32] | | 0.048 | | [1] | | | |
| Actinopterygii | [Gadiformes](http://en.wikipedia.org/wiki/Gadiformes) | Gadidae | *Melanogrammus aeglefinus* | | 3275.0 | [1] | | 6.0 | [33] | | 0.063 | | [1] | | | |
| Actinopterygii | [Ophidiiformes](http://en.wikipedia.org/wiki/Ophidiiformes) | Ophidiidae | *Acanthonus armatus* | | 180.0 | [34] | | 2.0 | [35] | | 0.027 | | [34] | | | |
| Actinopterygii | [Perciformes](http://en.wikipedia.org/wiki/Perciformes) | [Carangidae](http://en.wikipedia.org/wiki/Carangidae) | *Caranx bartholomoei* | | 4274.0 | [1] | | 27.0 | [32] | | 0.177 | | [1] | | | |
| Actinopterygii | [Perciformes](http://en.wikipedia.org/wiki/Perciformes) | [Carangidae](http://en.wikipedia.org/wiki/Carangidae) | *Caranx hippos* | | 2305.0 | [1] | | 27.0 | [32] | | 0.129 | | [1] | | | |
| Actinopterygii | [Perciformes](http://en.wikipedia.org/wiki/Perciformes) | [Istiophoridae](http://en.wikipedia.org/wiki/Istiophoridae) | *Tetrapturus audax* | | 2390.0 | [27] | | 23.2 | [36] | | 0.059 | | [27] | | | |
| Actinopterygii | [Perciformes](http://en.wikipedia.org/wiki/Perciformes) | Labridae | *Lachnolaimus maximus* | | 480.0 | [1] | | 25.5 | [37] | | 0.190 | | [1] | | | |
| Actinopterygii | [Perciformes](http://en.wikipedia.org/wiki/Perciformes) | [Lutjanidae](http://en.wikipedia.org/wiki/Lutjanidae) | *Lutjanus analis* | | 2490.0 | [1] | | 21.7 | [38] | | 0.084 | | [1] | | | |
| Actinopterygii | [Perciformes](http://en.wikipedia.org/wiki/Perciformes) | [Lutjanidae](http://en.wikipedia.org/wiki/Lutjanidae) | *Ocyurus chrysurus* | | 255.0 | [1] | | 27.0 | [39] | | 0.369 | | [1] | | | |
| Actinopterygii | [Perciformes](http://en.wikipedia.org/wiki/Perciformes) | Percidae | *Perca flavescens* | | 192.0 | [1] | | 21.8 | [31] | | 0.176 | | [1] | | | |
| Actinopterygii | [Perciformes](http://en.wikipedia.org/wiki/Perciformes) | Scombridae | *Euthynnus alletteratus* | | 6291.0 | [1] | | 24.0 | [36] | | 0.057 | | [1] | | | |
| Actinopterygii | [Perciformes](http://en.wikipedia.org/wiki/Perciformes) | [Scombridae](http://en.wikipedia.org/wiki/Scombridae) | *Katsuwonus pelamis* | | 7530.0 | [27] | | 23.0 | [36] | | 0.054 | | [27] | | | |
| Actinopterygii | [Perciformes](http://en.wikipedia.org/wiki/Perciformes) | [Scombridae](http://en.wikipedia.org/wiki/Scombridae) | *Scomberomorus maculatus* | | 765.0 | [1] | | 24.0 | [40] | | 0.084 | | [1] | | | |
| Actinopterygii | [Perciformes](http://en.wikipedia.org/wiki/Perciformes) | [Scombridae](http://en.wikipedia.org/wiki/Scombridae) | *Thunnus thynnus* | | 5210.0 | [1] | | 20.8 | [36] | | 0.059 | | [1] | | | |
| Actinopterygii | [Perciformes](http://en.wikipedia.org/wiki/Perciformes) | [Serranidae](http://en.wikipedia.org/wiki/Serranidae) | *Mycleroperca bonaci* | | 2712.0 | [1] | | 25.5 | [41] | | 0.073 | | [1] | | | |
| Actinopterygii | [Perciformes](http://en.wikipedia.org/wiki/Perciformes) | [Sphyraenidae](http://en.wikipedia.org/wiki/Sphyraenidae) | *Sphyraena barracuda* | | 9130.0 | [1] | | 24.4 | [42] | | 0.069 | | [1] | | | |
| Actinopterygii | [Perciformes](http://en.wikipedia.org/wiki/Perciformes) | Xiphiidae | *Xiphias gladius* | | 8800.0 | [27] | | 19.0 | [36] | | 0.022 | | [27] | | | |
| Actinopterygii | [Salmoniformes](http://en.wikipedia.org/wiki/Salmoniformes) | Salmonidae | *Coregonus artedi* | | 162.1 | [1] | | 14.2 | [31] | | 0.185 | | [1] | | | |
| Actinopterygii | [Salmoniformes](http://en.wikipedia.org/wiki/Salmoniformes) | Salmonidae | *Coregonus clupeaformis* | | 798.6 | [1] | | 12.7 | [31] | | 0.074 | | [1] | | | |
| Actinopterygii | [Salmoniformes](http://en.wikipedia.org/wiki/Salmoniformes) | Salmonidae | *Salmo salar* | | 5412.0 | [1] | | 15.3 | [31] | | 0.026 | | [1] | | | |
| Actinopterygii | [Salmoniformes](http://en.wikipedia.org/wiki/Salmoniformes) | Salmonidae | *Salmo trutta* | | 292.0 | [1] | | 16.0 | [31] | | 0.195 | | [1] | | | |
| Actinopterygii | [Salmoniformes](http://en.wikipedia.org/wiki/Salmoniformes) | Salmonidae | *Salmono gairdneri* | | 2750.0 | [1] | | 16.5 | [31] | | 0.030 | | [1] | | | |
| Actinopterygii | [Salmoniformes](http://en.wikipedia.org/wiki/Salmoniformes) | Salmonidae | *Salvelinus fontinalis* | | 255.0 | [1] | | 16.0 | [31] | | 0.172 | | [1] | | | |
| Actinopterygii | [Salmoniformes](http://en.wikipedia.org/wiki/Salmoniformes) | Salmonidae | *Salvelinus namaycush* | | 3240.0 | [1] | | 11.7 | [43] | | 0.041 | | [1] | | | |
| Actinopterygii | [Tetraodontiformes](http://en.wikipedia.org/wiki/Tetraodontiformes) | [Balistidae](http://en.wikipedia.org/wiki/Balistidae) | *Balistes capriscus* | | 295.0 | [1] | | 25.0 | [44] | | 0.296 | | [1] | | | |
|  |  |  |  | |  |  | |  |  | |  | |  | | | |
|  |  |  |  |  | |  | | | |  | |  | |  |  |  |

REFERENCES

1. Crile G, Quiring DP. 1940. A record of the body weight and certain organ and gland weights

of 3690 animals. *The Ohio Journal of Science* 40:219-285.

2. Clarke A, Rothery P. 2008. Scaling of body temperature in mammals and birds. *Functional Ecology* 22(1):58-67.

3. Vinogradov AE, Anatskaya OV. 2006. Genome size and metabolic intensity in tetrapods: a tale of two lines. *Proceedings of the Royal Society B* 273:27-32.

4. Carmena-Suero A, Siret JR, Caixejas, J, Arpones-Carmena D. 1980. Blood volume in male *Hyla septentrionalis* (tree frog) and Rana catesbeiana (bullfrog). *Comparative Biochemistry and Physiology Part A: Physiology* 67(1):187-189.

5. Roth G, Blanke J, Wake, D. 1994. Cell size predicts morphological complexity in the brains of frogs and salamanders. *Proceedings of the National Academy of Sciences* 91:4796-4800.

6. Taylor G, Nol, E, Boire D. 1995. Brain regions and encephalization in anurans: Adaptation or stability? *Brain Behavior and Evolution* 45:96-109.

7. Brattstrom BH. 1963. A preliminary review of the thermal requirements of amphibians. *Ecology* 44(2):238-255.

8. Zhang L, Lu X. 2012. Amphibians live longer at higher altitudes but not at higher latitudes. *Biological Journal of the Linnean* *Society* 106:623-632.

9. Sabat P, Riverosa, JM, Lopez-Pinto, C. 2005. Phenotypic flexibility in the intestinal enzymes of the African clawed frog *Xenopus laevis*. *Comparative Biochemistry and Physiology* 140:135-139.

10. Dawson WR. 1975. On the physiological significance of the preferred body temperatures of reptiles. In Gates DM (ed.) *Perspectives of Biophysical Ecology*, New York, Springer-Verlag, 443-473.

11. Köhler A, Sadowska J, Olszewska J, Trzeciak P, Berger-Tal O, Tracy CR. 2011. Staying warm or moist? Operative temperature and thermal preferences of common frogs (*Rana* *temporaria*), and effects on locomotion. *Herpetological Journal* 21:17-26.

12. Seebacher F, Elsey RM, Trosclair PL 3rd. 2003. Body temperature null distributions in reptiles with nonzero heat capacity: seasonal thermoregulation in the American alligator (*Alligator mississippiensis*). *Physiological and Biochemical Zoology*  76(3):348-359.

13. Lang J. 1979. Thermophilic response of the American alligator and the American crocodile to feeding. *Copeia* 1:48-59.

14. McGinnis SM, Moore RG. 1969. Thermoregulation in the Boa constrictor. *Herpetologica* 25(1): 38-45.

15. Brattstrom BH. 1965. Body temperatures of reptiles. *American Midland Naturalist* 73(2):376-422.

16. Isaac LA, Gregory PT. 2004. Thermoregulatory behaviour of gravid and non-gravid female grass snakes (*Natrix natrix*) in a thermally limiting high-latitude environment. *Journal of Zoology, London* 264:403-409.

17. Mushinsky H, Hebrard J, Walley M. 1980. The role of temperature on the behavioral and ecological associations of sympatric water snakes. *Copeia* 4:744-754.

18. Bartholomew G, Lasiewski R. 1965. Heating and cooling rates, heart rate and simulated diving in the Galapagos marine iguana. *Comparative Biochemistry and Physiology* 16(4):573-582.

19. Clusella-Trullas S, Blackburn TM, Chown SL. 2011. Climatic predictors of temperature performance curve parameters in ectothermy imply complex responses to climate change. *The American Naturalist* 177(6):738-751.

20. Van Mierop L, Barnard S. 1978. Further Observations on Thermoregulation in the Brooding Female *Python molurus bivittatus* (Serpentes: Boidae). *Copeia* 4: 615-621.

21. Heath M, McGinnis SM. 1980. Body Temperature and Heat Transfer in the Green Sea Turtle, *Chelonia mydas*. *Copeia* 4:767-773.

22. Brown G, Brooks R. 1991. Thermal and Behavioral Responses to Feeding in Free-Ranging Turtles, *Chelydra serpentina*. *Journal of Herpetology* 25(3): 273-278.

23. Howey C, Dinkelacker S. 2009. Habitat Selection of the Alligator Snapping Turtle (*Macrochelys temminckii*) in Arkansas. *Journal of Herpetology* 43(4) 589-596.

24. Yagi KT, Litzgus, JD. 2013. Thermoregulation of Spotted Turtles (*Clemmys guttata*) in a Beaver-Flooded Bog in Southern Ontario, Canada. *Journal of Thermal Biology* 38(5): 205-213.

25. Lapid, R, Nir I, Snapir N, Robinzon B. 2004. Reproductive traits in the spur-thighed tortoise (*Testudo graeca terrestris*): new tools for the enhancement of reproductive success and survivorship. *Theriogenology* 61(6):1147-1162.

26. Heithaus M, 2001. The biology of tiger sharks, *Galeocerdo cuvier*, in Shark Bay, Western Australia: sex ratio, size distribution, diet, and seasonal changes in catch rates. *Environmental Biology of Fishes* 61:25–36.

27. Lisney T, Collin S. 2009. Brain morphology in large pelagic fishes: a comparison between sharks and teleosts. *Journal of Fish* *Biology* 68(2):532-554.

28. Kohler NE, Turner PA, Hoey JJ, Natanson LJ, Briggs R. 2002. Tag and recapture data for three pelagic shark species: Blue shark (*Prionace glauca*), Shortfin Mako (*Isurus xyrinchus*) and Porbeagle (*Lamna nasus*) in the North Atlantic Ocean.

*International Commission for the Conservation of Atlantic Tuna* (ICCAT) 54(4):1231-1260.

29. Smale M. 2002. Occurrence of *Carcharias taurus* in nursery areas of the Eastern and Western Cape, South Africa. *Marine and* *Freshwater Research* 53:551-556.

30. Wallman H, Bennett W. Effects of parturition and feeding on thermal preference of Atlantic stingray, *Dasyatis sabina* (Lesueur). *Environmental Biology of Fishes* 75:259-267.

31. Jobling M. 1981. Temperature tolerance and the final preferendum-rapid methods for the assessment of optimum growth

temperatures. *Journal of Fish Biology* 19:439-455.

32. Palomares M, Pauly D. 1989. A multiple regression model for predicting the food consumption of marine fish populations. *Australian Journal of Marine and Freshwater Research* 40: 259-284.

33. Scott JS. 1982. Depth, Temperature and Salinity Preferences of Common Fishes of the Scotian Shelf. *Journal of Northwest* *Atlantic Fisheries Science* 3:29-39.

34. Fine ML, Horn MH, and Cox B. 1987. *Acanthonus armatus*, a deep-sea teleost fish with a minute brain and large ears. *Proceedings of the Royal Society B* 230(1259):257-265.

35. Nielsen, JG. 1990. Ophidiidae. In, Quero JC (ed.) *Check-list of the fishes of the eastern tropical Atlantic (CLOFETA)* Lisbon, UNESCO, 564-573.

36. Boyce DG, Tittensor DP, Worm B. 2008. Effects of temperature on global patterns of tuna and billfish richness. *Marine Ecology Progress Series* 355:267-276.

37. Colin P. 1982. Spawning and Larval Development of the Hogfish, '*Lachnolaimus maximum*' (Pisces: Labridae). *Fishery Bulletin , NOAA* 80(4):1-10.

38. Rivas LR. 1970. Snappers of the Western Atlantic*. Commercial Fisheries Review* 32(1): 41.

39. Wallace RJ. 1977. Thermal acclimation, upper temperature tolerance, and preferred temperature of juvenile yellowtail, *Ocyurus chrysurus*. *Bulletin of Marine Science* 27(2):292-298.

40. Godcharles M, Murphy M. 1986. Species profiles: Life History and Environmental Requirements

of Coastal Fishes and Invertebrates (South Florida):King Mackeral and Spanish Mackeral. *US Fish and Wildlife Service, Biological Report* 82:1-18.

41. Koch V. 2011. The Spatial Ecology of Black Groupers (*Mycteroperca bonaci*) in the Upper Florida Keys, in Marine Biology and Fisheries. University of Miami.

42. O’Toole AC, Danylchuk AJ, Suski CD, Cooke SJ. 2010. Consequences of catch-and-release angling on the physiological status, injury, and immediate mortality of great barracuda (*Sphyraena barracuda*) in The Bahamas. *ICES Journal of Marine Science* 67:1667–1675.

43. Johnson J, Kelsch S. 1998. Effects of evolutionary thermal environment on temperature-preference relationships in fishes. *Environmental Biology of Fishes* 53:447-458.

44. Reynolds WW, Casterlin M. 1981. Thermoregulatory behavior of the triggerfish *Balistes fuscus* in an electronic shuttlebox. *Hydrobiologia* 83(2): 255-256.
